# Psychometric evaluation of the PROMIS® Depression Item Bank: an illustration of classical test theory methods

**DOI:** 10.1186/s41687-019-0127-0

**Published:** 2019-07-30

**Authors:** Sandra Nolte, Cheryl Coon, Stacie Hudgens, Mathilde G. E. Verdam

**Affiliations:** 1 Department of Psychosomatic Medicine, Center for Internal Medicine and Dermatology, Charité - Universitätsmedizin Berlin, Berlin, Germany; 2 Centre for Population Health Research, School of Health and Social Development, Deakin University, Geelong, Australia; 3 Outcometrix, 2912 NE Plaza Drive, Tucson, AZ 85716 USA; 4 Clinical Outcomes Solutions, 1820 East River Road, Suite 220, Tucson, AZ 85718 USA; 5 Department of Medical Psychology, Academic Medical Centre, University of Amsterdam, Amsterdam, The Netherlands; 6 Department of Methods & Statistics Institute of Psychology, Leiden University, P.O. Box 9555, 2300 RB Leiden, The Netherlands

**Keywords:** Classical test theory, Patient-reported outcomes, Validity, Reliability, Factor analysis, Structural equation modeling

## Abstract

**Background:**

Psychometric theory offers a range of tests that can be used as supportive evidence of both validity and reliability of instruments aimed at measuring patient-reported outcomes (PRO). The aim of this paper is to illustrate psychometric tests within the Classical Test Theory (CTT) framework, comprising indices that are frequently applied to assess item- and scale-level psychometric properties of PRO instruments.

**Methods:**

Using data on the PROMIS Depression Item Bank, typical CTT indices for the assessment of psychometric properties are illustrated, including content validity, item-level data exploration, reliability, and construct validity, particularly confirmatory factor analysis, to test the unidimensionality assumption underlying the item bank. Analyses are carried out on an original item set of 51 depression items, the final (official) PROMIS Depression Item Bank consisting of 28 items, and an 8-item short form.

**Results:**

The analyses reported provide an informative illustration on how item- and scale-level reliability and validity statistics can be used to assess the psychometric quality of a PRO instrument. The results illustrate how the reported statistics can be used for item selection from an item pool (*here*: 51 items). Both the (final) 28-item bank and the 8-item short form show good psychometric properties supporting the high quality of individual items and the unidimensionality assumption of the item bank.

**Conclusions:**

It is our hope that our illustration of CTT methods, in conjunction with two companion papers illustrating modern test theory methods, will help researchers to confidently apply a range of statistical tests to evaluate item- and scale-level psychometric performance of PRO instruments.

## Background

Test theory, also referred to as psychometric theory, is concerned with the theory of measurement of psychological constructs [1]. Although initial developments of test theory date back more than a century [2], psychometric theory is more topical than ever, in particular in the field of medicine. Over the past decade, the inclusion of the patient perspective in clinical care and research (e.g., through measurement of self-reported outcomes such as symptom burden, emotional, physical, and social functioning) has developed to be a necessary rather than merely desired aspect in the evaluation of treatment effectiveness, with regulatory agencies worldwide recommending the inclusion of patient-reported outcomes (PROs) in clinical trials [3–5]. The growing importance of patient-centeredness, not only in the delivery of healthcare but also in healthcare research, is further noticeable in the increased funding dedicated to both improvement and standardization of PRO measures. For example, the Patient-Centered Outcomes Research Institute (PCORI) was founded in the United States in 2010 with the aim to fund only those comparative effectiveness research studies that demonstrate engagement with and to be of relevance to patients and caregivers [6]. Further, the standardization of PRO assessment has become a major research area; initiatives, such as the Patient-Reported Outcomes Measurement Information System (PROMIS®), have been founded to develop and validate item banks on major health domains that are successively being implemented across the globe [7, 8].

In view of increased use and relevance of PROs, it is crucial that these self-reported outcomes are measured with the utmost precision. For this, psychometric theory is pivotal as it offers a range of tests that can be used as supportive evidence of both validity and reliability of a PRO instrument. In other words, because the psychological phenomenon of interest cannot be observed directly (e.g., depression), it is necessary to assess the extent to which the self-report measure (i.e., the set of items on a questionnaire) can be interpreted as a valid and reliable reflection of the construct that it is intended to measure. As such, psychometric theory plays an important role in the development of PRO instruments and the evaluation of their psychometric quality.

Both traditional and modern test theory methods can be employed to evaluate an instrument’s psychometric properties. At the core of both methods is that they are concerned with the measurement of unobservable (latent) constructs through a set of observed variables to get as best an approximation of the latent variable as possible. Traditional test theory, also referred to as Classical Test Theory (CTT), is the older of the two and still the most frequently applied method in health-related quality of life research; its use is also suggested by the U.S.-American Food and Drug Administration [4]. Generally, CTT includes indices that describe a PRO instrument’s validity (content/face, construct [structural, convergent, discriminant, and known groups], criterion [concurrent and predictive]) and its reliability (internal consistency and test-retest reliability) [9, 10].

The aim of this paper is to provide an illustration of a range of analyses within the CTT framework. We discuss both the (practical) advantages and disadvantages of the analyses and their interpretation. This educational paper is part of a series of papers initiated by the Psychometrics Special Interest Group (SIG) of the International Society for Quality of Life Research (ISOQOL) aimed at introducing different psychometric techniques to analyze item properties of a PRO instrument, i.e., CTT as presented in this paper, item response theory (IRT) [Stover et al, copublished in this issue], and Rasch measurement theory (RMT) [Cleanthous et al, copublished in this issue] methods. To outline the methods used to perform psychometric tests applying a CTT-based approach, the PROMIS® Emotional Distress - Depression Item Bank version 1.0 was selected because of its availability and extensive use since its development in 2011 [11]. Although other PRO instruments are available to assess depression (e.g., Center for Epidemiological Studies – Depression [CES-D] [12], Patient Health Questionnaire [PHQ-9] [13], Beck Depression Inventory II [BDI-II)] [14]), the PROMIS Depression Item Bank has been shown to provide more information than conventional measures for which these short-form measures are comparable [11]. The objective of the present paper is to use data on the PROMIS Depression Item Bank to demonstrate how CTT methods may be employed to evaluate the psychometric properties of a set of PRO items.

## Methods

### PROMIS emotional distress - Depression Item Bank version 1.0

The PROMIS Depression Item Bank was developed following a comprehensive literature search and qualitative methods, which resulted in an initial pool of 518 items. Using psychometric analyses, these were subsequently reduced to a preliminary pool of 56 items for calibration testing. After thorough quantitative analyses, the final PROMIS Depression Item Bank contains 28 items [11]. The items included in the final bank specifically focus on negative mood, decreases in positive emotions, cognitive deficits, negative self-image, and negative social cognition [7]. The items are scored on a 5-point verbal response scale (i.e., ordered categorical item responses) where respondents are asked to rate the experienced frequency of symptoms (*never*, *rarely*, *sometimes*, *often*, *always*).

For the purpose of this series of papers, a subset of the PROMIS calibration samples was made available by the PROMIS Health Organization. This dataset comprised 51 of the 56 preliminary PROMIS depression items, and was seen as a valuable resource in the public domain by aforementioned ISOQOL Psychometrics SIG to compare item performance results from CTT, IRT, and RMT methods. The full sample data is also publicly available (see 10.7910/DVN/0NGAKG).

The PROMIS Calibration Studies sample included 21,133 respondents, with *n* = 1532 recruited from primary research sites associated with PROMIS network sites, while the vast majority (*n* = 19,601) was recruited from an Internet polling company; further details about the sampling are available in the introductory paper to this special issue [ref]. For the purpose of illustrating different methods to assess the psychometric properties, we only used data from respondents from the general population that were administered the full item bank (*n* = 925), and excluded respondents that were flagged by predetermined speed-of-response criteria (*n* = 100) and who had missing item responses (*n* = 72). This resulted in a total sample of *N* = 753 (see Table 1 for an overview of demographic information). Pilkonis et al. [11] have previously described results of psychometric analyses on the same data for the purpose of item selection. The current paper does not have such a substantive aim – we do not wish to add to the analyses reported of Pilkonis et al. [11] – but rather our aim is to use these data for illustrative purposes to introduce CTT methods. As the final item bank contains 28 items, which can further be applied as one of the many PROMIS depression short forms (in this case, 8-item Short Form 8b), subsequent analyses were carried out on three item subsets, i.e. 51, 28, and 8 items, respectively.

**Table 1:** Demographic Characteristics of the PROMIS Sample (*N* = 753)

	General population sample (*N* = 753)
*Age; Mean (SD)*	51 (19)
*Age group; N (%)*	
18–35	204 (27)
36–50	164 (22)
51–65	182 (24)
66–88	198 (26)
*Gender; N (%)*	
Female	391 (52)
Male	361 (48)
*Ethnicity; N (%)*	
Caucasian	597 (79)
African-American	73 (10)
Other	83 (11)
*Education; N (%)*	
Primary	20 (2)
Secondary	149 (20)
Post-secondary	346 (46)
Tertiary	238 (32)
*Relationship status; N (%)*	
Single	120 (16)
Married or with relationship	485 (64)
Separated or divorced	87 (11)
Widowed	59 (8)

### Content/face validity

Content validity refers to the extent to which a questionnaire’s items reflect the content of the construct to be measured. Establishing content validity is a theoretical and subjective undertaking that is part of the instrument development process; although it is an important psychometric quality it does not require statistical evaluation. It is done by providing a definition of the target construct (e.g., using literature search, focus groups, interviews) and subsequent development of new items and/or selection of items from existing instruments. A related concept is face validity, which refers to the extent to which experts agree on what the instrument *appears* to measure. The main distinction between the two is that content validity refers to the instrument development process, whereas the latter term is usually used in the context of critical review of existing instruments [15].

### Data exploration at the item level

Although CTT techniques generally focus on tests at the scale level, it is useful to include item-level exploration in the evaluation of an instrument’s measurement properties. This can be done by, for example, inspecting frequency distributions (ordinal item responses) or means and variances (continuous item responses). Generally, variability across response categories and items is desirable as it indicates that respective item’s content is relevant to respondents and the response categories are appropriate for determining the continuum of a psychological construct. The distribution of responses also gives insight into potential floor or ceiling effects. While item-level exploration can give important insight into the quality of individual items, strict decision rules regarding whether response variability is adequate is difficult given that frequency distributions/means (variances) are dependent on the construct being measured and the sample used. For example, in a general population sample, response variability on items about severe depression symptoms is expected to be limited as compared to mild depression symptoms, whereas one may expect the reverse in a clinical sample. Additionally, a clinical sample participating in a clinical trial is likely to demonstrate quite different item response distributions at baseline (i.e., when they are symptomatic and in need of treatment) versus post-treatment (i.e., when the treatment has hopefully improved symptoms). Therefore, contextual factors should be considered when interpreting item-level analyses; one may accept different distributions in different samples under different conditions as reasonable. Given the ordinal scaling of the PROMIS depression items, in this paper the frequency distribution for each item was examined to evaluate data completeness, potential floor and/or ceiling effects, and the variability of responses across categories.

Item discrimination refers to the extent to which an item measures the underlying construct of interest, and thus is able to discriminate between respondents. Item discrimination is determined by exploring the correlation of an individual item with the whole item set (item-total correlation) or with all other items of the set (corrected item-total correlation). In this paper, we considered corrected item-total correlations of *r*_*itc*_ < 0.4 as the cut-off following the developers of the PROMIS Depression Item Bank [11], but other cut-offs have been suggested (see [16]).

Aforementioned distinction regarding whether items are scored on an ordinal (e.g., Likert) or continuous response scale also influences the choice of further statistical methods used to inspect an instrument’s quality. As the former type can only take on a limited number of values, a decision has to be made about whether these can be treated as (approximations of) continuous item responses or as ordered categories. Inspection of skewness/kurtosis statistics (transformed to a *z*-score) and normality tests (e.g., Kolmogorov-Smirnov test, Shapiro-Wilk test) can be used to evaluate the assumption of normal distribution of item scores. However, with larger samples (*n* > 200) these tests can turn out to be significant even with only small deviations from normality. As an alternative, visual inspection of the distribution or substantive considerations may be more appropriate to guide a decision regarding which statistical methods to use [17].

### Reliability

Reliability refers to the extent to which the scores on an item set reflect the ‘true’ score on the construct of interest. Scores that are highly reliable are accurate, reproducible, and consistent reflections of the underlying construct that the item set measures. Different methods exist to evaluate scale reliability where the reliability coefficient reflects the proportion of true variance in the variance of the observed scores, with higher values indicating a more reliable estimate of the true scores.

The most well-known and widely applied reliability coefficient is Cronbach’s alpha [18], also referred to as a measure of internal consistency. Values > 0.70 are generally taken to indicate good reliability; however, the appropriateness of this – or any – threshold may vary depending on the purpose of the instrument [19]. Although Cronbach’s alpha is most often used as a reliability coefficient, it is not without critique [20–22]. In particular, it is estimated under the assumption that all items are equally good measures of the construct (i.e., they are essentially tau equivalent) and violation of this assumption may lead to an underestimation of the reliability. Moreover, its calculation is influenced by the number of items of the test and the average interrelatedness between the items, which may result in high reliability estimates for longer tests regardless of whether the items measure a homogeneous (i.e., unidimensional) construct or not. Therefore, interpretation of Cronbach’s alpha should coincide with a careful consideration of both the instrument’s content and number of items in the scale. In order to take into account deviating distributional properties, it may be more appropriate to apply alternatives that have been developed, such as a special correction to the reliability coefficient that has been suggested for the ordinal case [23] and the Kuder-Richardson Formula (K-R20) that is a simplified version of Cronbach’s alpha for the dichotomous case [24]. The internal consistency of the PROMIS Depression Item Bank was assessed using the alpha coefficient with a threshold criterion of > 0.70 [16].

### Construct validity

Construct validity refers to the extent to which the behavior of the instrument’s scores are consistent with what would be expected from the construct of interest. This can be evaluated by looking at internal relationships, relationships to scores of other instruments or differences between relevant groups.

In the following we address construct validity in terms of internal relationships between variables (i.e., dimensionality/structural validity) using factor analysis [10]. Further analyses of construct validity are considered outside the scope of the current article. As an example, in the context of the PROMIS Depression Item Bank one could consider further investigation of construct validity by looking at the correlations with different measures of depression, or investigate differences in depression scores between a clinical sample and general population sample.

### Dimensionality

Assessment of an instrument’s dimensionality is also referred to as structural validity. It is used to assess the degree to which the scores of an item set are an adequate reflection of the dimensionality of the construct. Within the CTT framework, structural validity is usually assessed using factor analysis. The factor analytic framework is historically closely connected to the CTT framework, although it can be considered as a more ‘modern’ set of statistical techniques as it allows for the investigation of item- and scale-characteristics of an instrument using less restrictive assumptions. That is, the flexibility of the factor analytic framework can be used to model the individual item characteristics without imposing equality restrictions (i.e., assuming tau equivalences or parallelism of the items).

Factor analysis is a group-level analysis technique aimed at attributing sets of observed variables to one or more latent variables [25]. While exploratory factor analysis is generally used when the relationship among the variables is unknown and the researcher is seeking dimensionality insight from the analysis, confirmatory factor analysis (CFA) is more appropriate when relationships among the variables are already hypothesized (e.g., via a conceptual framework used to construct the instrument). In CFA, the more variance of an item can be explained by the hypothesized latent variable (factor), the better the item fits to the construct. This is usually expressed in terms of *factor loadings*, with the squared loading indicating the variance explained. Loadings > 0.50 are deemed the minimum; loadings > 0.70 are desirable [19]. To confirm the hypothesized one-factor structure of the PROMIS Depression Item Bank, in this paper unidimensional CFA was used to assess the degree to which it is appropriate to combine the 51, 28, and 8 items, respectively, in one domain [26].

To evaluate how well the hypothesized model fits the data the most widely used method is maximum likelihood (ML) estimation. It is valid under the assumption that observed scores follow a multivariate normal distribution; however, alternative estimation methods are required when this assumption is not met (e.g., with ordinal data [27, 28]). Options for ordinal data include weighted least squares for large samples and simple models, and robust (or diagonally) weighted least squares (WLSMV/DWLS) for smaller samples and complex models. These estimation methods use an asymptotic covariance weight matrix to adjust for the non-normality of ordinal data [29]. In addition, estimation methods for ordinal data usually require adjustments to the input matrices of variances and covariances. That is, in the ordinal and dichotomous case, polychoric and tetrachoric correlations, respectively, are estimated instead.

To evaluate overall goodness-of-fit, the χ^2^ test of exact fit [30] can be used where a significant χ^2^ value indicates a significant difference between data and model [26]. As this value is dependent on sample size and number of model parameters included [26, 31], alternative fit indices have been developed. A prominent approximate fit index is the root mean square error of approximation (RMSEA), with RMSEA≤.05 indicating close and RMSEA≤.08 indicating reasonable approximate fit [32]. Finally, incremental fit indices are used to compare the model to an alternative or baseline model [33], with the comparative fit index (CFI) [34] most frequently recommended [35]. CFI values range between 0 and 1 [19, 34]; CFI ≥ 0.95 is indicative of good model fit [36]. The Tucker Lewis Index (TLI) – or non-normed fit index (NNFI) – is conceptually similar to CFI. While not normed between 0 and 1, values close to 1 are considered to indicate good fit [19]. For more detailed overviews of different fit indices and their interpretation, the reader may be referred elsewhere [36–38].

In this paper, CFA was conducted using polychoric correlations and WLSMV with robust standard errors and a mean- and variance-adjusted test statistic (using a scale-shifted approach). Further, above fit indices based on the adjusted chi-square test statistic were considered to interpret goodness of model fit. In the event that the unidimensional model did not provide acceptable model fit, modification indices (i.e., the expected change in model fit if specific model revisions were made) and residual correlations (i.e., the excess relationship between items after accounting for the underlying factor) were inspected to identify reasons for model misfit [39]. Analyses were conducted with the package Lavaan that runs in the freely available R software [40]. Syntaxes of the analyses are available on request.

## Results

### Content/face validity

As content validity of the PROMIS Depression Item Bank was performed by the original developers, it is only presented here for completeness [41]. It comprised a comprehensive literature search [41] and focus groups with patients to ensure that the instrument reflected the perspectives of the population of interest [42]. Moreover, selection of items was (partly) based on content balancing to retain a representative group of symptoms and complaints in the final bank. Face validity was assessed by asking experts to review the resulting bank, and to define and describe the content that was being measured [43].

### Data exploration at the item level

Normality tests showed that all items deviated significantly from normality, with severe right skewness (Table 1). Visual inspection of histograms and frequency distributions confirmed that response options ‘never’ and ‘rarely’ were chosen more frequently (e.g., regularly > 60% of respondents) than the other response options. It needs to be taken into account that the sample used consisted of a representative population sample, which may explain the relatively low percentages of endorsement of the more extreme response categories. The finding that these items show low response variability is thus limited for administration in a general population sample, as the behavior of items could be quite different in other, e.g. clinical, samples. Dependent on the intended use of the instrument, these results could serve as basis for item selection by removing items that show the most skewed item response distributions (as was also done by Pilkonis et al. [11]). For example, one could remove item 1 (‘I reacted slowly to things that were said or done’) as only 3.5% fall within the two highest response categories. Alternatively, item 15 (‘I disliked the way my body looked’) seems to show almost a uniform response distribution, with similar percentages of respondents in each response category. This pattern of responses deviates from the other response patterns and may indicate that this item measures something else than depression (as measured by the other items). In contrast, an item such as item 32 ‘I wished I were dead and away from it all’, where 84% of respondents chose response option ‘never’ and only 1% indicated ‘always’, could be retained based on item content, as it could be deemed a relevant item to cover suicidal thoughts. However, if the instrument was to be used in a clinical population, then we may want to be more conservative with item reduction, as these items with low endorsement in a general population may be relevant in a clinical population and important for measuring severe conditions.

Based on these distributional results, taken in combination with the nature of the response options (i.e., frequencies), we decided that the PROMIS depression items should be treated as ordinal, although observed variables with five response categories are sometimes treated as continuous.

The corrected item-total correlations were investigated next (three rightmost columns of Table 1) using polychoric correlations to take into account the ordinal nature of the data, as suggested by Gaderman, Guhn and Zumbo [23]. There was one item with correlation smaller than 0.40 (i.e., item 49 ‘I lost weight without trying’), which could therefore be considered for removal. As all other corrected item-total correlations were rather high, one could consider using more stringent criteria for item selection. For example, items 11, 15 and 53 could be removed based on the criterion that the corrected item-total correlation should be larger than 0.70, and an additional 8 items would be candidates for removal if the criterion were increased to 0.75 (items 1, 3, 18, 20, 24, 34, 37, and 43). These candidate items for removal are consistent with the item selection of Pilkonis et al. [11]). Thus, item-level data exploration can provide valuable information about the performance of items within a scale; however, it should be used in combination with further substantive considerations to retain a sensible item set.

### Reliability

The reliability coefficient was calculated based on polychoric correlations, as appropriate for ordinal data [23]. The alpha reliability coefficient was high, with 0.989 for the total item bank (51 items), 0.988 for the final item bank (28 items), and 0.974 for the 8-item short form, with the latter finding indicating that reliable measurement of depression can be attained with a relatively small number of items. To consider item reduction based on the reliability coefficient, one could further inspect the alpha-if-item-deleted statistic (i.e., the expected alpha of the instrument when the specific item is deleted), which identifies items that may not be highly related to the other items and the domain of interest (depression). For example, the deletion of aforementioned item 49 from the 51-item bank would not substantially change alpha (0.990 versus 0.989), suggesting that this item was somewhat different from the other 50 items.

### Construct validity - dimensionality

The one-factor solution for the 51-item bank showed acceptable model fit (Table 2). All standardized factor loadings were quite high (mostly > 0.7; Table 3), thus supporting the unidimensionality assumption of the underlying depression factor. Only for item 49 the factor loading was low; hence, again a candidate item for deletion. Using more stringent criteria, e.g. factor loadings of ≥0.7, there are three additional candidate items for deletion. Inspection of modification indices showed that the three most problematic instances of misfit in the model were a result of high residual correlations between items 16 (‘I felt like crying’) and 34 (‘I had crying spells’), items 3 (‘I felt that I had no energy’) and 18 (‘I got tired more easily than usual’), and items 32 (‘I wished I were dead and away from it all’) and 39 (‘I felt I had no reason for living’). These results indicate that, in this general population, the respective relationship between these items was not well explained by the underlying factor. In other words, these items have something else in common besides what is captured by the depression factor. Closer inspection of respective content suggests that each item pair seem to measure similar symptoms. Such finding could be an indication of multidimensionality as items that belong to the same subdomain could be considered to reflect a multidimensional depression construct; alternatively, it could be an indication of item redundancy, i.e., keeping both items of respective item pair in the bank does not add substantial new information so that one item may be removed. This hypothesis could be further explored either by looking at patterns of residual correlations or by fitting multidimensional factor models.

**Table 2:** Model-fit results of confirmatory factor analysis (CFA) of extended item bank (51 items), final item bank (28 items) and short-form 8b (8 items) of the PROMIS Depression Item Bank using WLSMV^a,b^

Model	χ^2^ value	df	*p*	RMSEA [90% CI]	CFI	TLI
Unidimensional model with 51 items	5729.6	1224	<.001	0.070 [0.068; 0.072]	0.953	0.951
Unidimensional model with 28 items	1473.27	350	<.001	0.065 [0.062; 0.069]	0.983	0.982
Unidimensional model with 8 items	177.71	20	<.001	0.101 [0.088; 0.115]	0.995	0.992

^a^WLSMV: robust weighted least squares

^b^Sample size for the models with 51, 28 and 8 items, respectively, was N = 753

**Table 3:** Factor loadings for the unidimensional factor models of the PROMIS Depression Item Bank

51 items	Factor loading	Residual variance	28 items	Factor loading	Residual variance	8 items	Factor loading	Residual variance
EDDEP01	0.733	0.462	EDDEP04	0.928	0.139	EDDEP04	0.924	0.147
EDDEP03	0.763	0.418	EDDEP05	0.9019	0.188	EDDEP05	0.888	0.212
EDDEP04	0.922	0.151	EDDEP06	0.901	0.189	EDDEP06	0.910	0.172
EDDEP05	0.893	0.203	EDDEP07	0.825	0.319	EDDEP17	0.891	0.206
EDDEP06	0.894	0.200	EDDEP09	0.900	0.190	EDDEP22	0.905	0.180
EDDEP07	0.826	0.317	EDDEP14	0.852	0.274	EDDEP29	0.900	0.190
EDDEP08	0.793	0.371	EDDEP17	0.877	0.232	EDDEP36	0.925	0.145
EDDEP09	0.897	0.195	EDDEP19	0.904	0.182	EDDEP41	0.944	0.109
EDDEP11	0.530	0.719	EDDEP21	0.846	0.284			
EDDEP12	0.796	0.366	EDDEP22	0.918	0.158			
EDDEP14	0.843	0.289	EDDEP23	0.823	0.323			
EDDEP15	0.609	0.629	EDDEP26	0.868	0.247			
EDDEP16	0.820	0.328	EDDEP27	0.870	0.243			
EDDEP17	0.872	0.239	EDDEP28	0.823	0.323			
EDDEP18	0.770	0.407	EDDEP29	0.898	0.194			
EDDEP19	0.901	0.188	EDDEP30	0.802	0.357			
EDDEP20	0.773	0.403	EDDEP31	0.863	0.254			
EDDEP21	0.835	0.304	EDDEP35	0.861	0.259			
EDDEP22	0.904	0.183	EDDEP36	0.911	0.169			
EDDEP23	0.816	0.335	EDDEP39	0.879	0.226			
EDDEP24	0.710	0.496	EDDEP41	0.938	0.120			
EDDEP26	0.853	0.272	EDDEP42	0.799	0.362			
EDDEP27	0.861	0.258	EDDEP44	0.827	0.317			
EDDEP28	0.813	0.339	EDDEP45	0.832	0.308			
EDDEP29	0.901	0.189	EDDEP46	0.890	0.308			
EDDEP30	0.826	0.318	EDDEP48	0.880	0.226			
EDDEP31	0.848	0.281	EDDEP50	0.795	0.368			
EDDEP32	0.891	0.206	EDDEP54	0.859	0.261			
EDDEP33	0.817	0.332						
EDDEP34	0.779	0.394						
EDDEP35	0.863	0.255						
EDDEP36	0.900	0.190						
EDDEP37	0.726	0.472						
EDDEP38	0.802	0.356						
EDDEP39	0.904	0.183						
EDDEP40	0.841	0.293						
EDDEP41	0.927	0.140						
EDDEP42	0.792	0.373						
EDDEP43	0.776	0.398						
EDDEP44	0.838	0.298						
EDDEP45	0.835	0.303						
EDDEP46	0.819	0.330						
EDDEP47	0.800	0.360						
EDDEP48	0.871	0.241						
EDDEP49	0.300	0.910						
EDDEP50	0.788	0.380						
EDDEP52	0.838	0.298						
EDDEP53	0.614	0.623						
EDDEP54	0.874	0.236						
EDDEP55	0.860	0.260						
EDDEP56	0.846	0.283						

Results of the CFA containing the 28 items of the final PROMIS Depression Item Bank generally supported the unidimensionality assumption as did the 8-item short form, with the latter showing somewhat less optimal model fit (Table 2). Further, standardized factor loadings for all items were very high, with associated low proportions of unexplained item variances (Table 3).

## Discussion

This paper aimed to demonstrate how CTT methods may be employed to evaluate the psychometric properties of a set of PRO items. It is part of a series of papers initiated by the ISOQOL Psychometrics SIG, which was aimed at comparing CTT with two modern test theory approaches, i.e. IRT and RMT methods, with each using the same dataset of PROMIS depression items.

First, item-level analyses were carried out. As the data made available were collected from a general population, the prevalence of the various depression symptoms was rather low, which resulted in high floor effects for many of the items; hence, there was a high propensity for item response distributions to be skewed towards the bottom category. Such response distribution, however, would be expected for a general population sample and was not deemed undesirable in this specific context. The items with the greatest floor effects (e.g., percentage in category 1 > 80%) were from the suicidality subdomain. Endorsement of these items would indicate the most severe levels of depression, so this pattern was consistent with the expected endorsement rate. In fact, only 10 of the 51 items did not display severe floor effects. These 10 items spanned four of the six subdomains (i.e., mood, cognition, behavior, somatic complaints), so it was likely that these items were indicators of milder depression levels as opposed to the items displaying floor effects that would be more appropriate (and necessary) for measuring moderate and severe depression levels. Keeping these limitations in mind (i.e., using data from a non-clinical sample), for the purpose of this paper, proposed strategies for item reduction focused on item redundancy as well as weak relationships of each item with the other items. Balancing these decisions with respective item content, we identified several candidate items for deletion that were largely consistent with those identified by Pilkonis et al. [11]. However, one should take into account that the reliability and validity of the final instrument is limited to a population sample, and thus cannot be readily applied to other – e.g. clinical – samples.

Second, the alpha reliability coefficient showed high internal consistency of all three instruments, i.e. the 51-, 28-, and 8-item version, respectively. However, these results need to be interpreted with care. As the reliability coefficient increases as the number of items increases, the interpretation of alpha of as many as 51 items – or 28 items – may not be useful as the value can be an artifact of the large number of items included. Nevertheless, it is reassuring that the 8-item short form showed a reliability of > 0.90, supporting the notion of good reliability of these PROMIS depression items [15]. One should keep in mind that good reliability can be achieved at the expense of item content, e.g. combining very similar but not necessarily complimentary items into one scale will result in highly reliable but not necessarily very valid scales. Thus, the development of an informative measurement scale requires a careful consideration of both reliability and validity.

Finally, the results of the confirmatory factor analyses showed satisfactory fit for all three instruments. Hence, there was sufficient support that the PROMIS depression items are indeed unidimensional. In other words, there was insufficient support for the alternative hypothesis, that is, that the items belonging to respective predefined subdomains (negative mood, decreases in positive emotions, cognitive deficits, negative self-image, and negative social cognition [7]) might be sufficiently different from items from other subdomains to justify a multidimensional depression construct. Nevertheless, the interested researcher could have considered alternative models, including multidimensional models or higher-order factor models, to investigate the tenability of such theoretical structures. In addition, more restricted models could have been considered to test specific substantive hypotheses. For example, one could test whether the individual factor loadings can be constrained to equality to test the tenability of the tau equivalence assumption. We chose to illustrate the application of a confirmatory unidimensional factor model with freely estimated factor loadings to illustrate the potential of this type of analysis. The flexibility of the factor analytic framework can further be used to impose restrictions on (individual) model parameters to test (further) substantive hypotheses. One could, for example, investigate possible differences in the factor structure across groups of participants or across time (i.e., using the measurement invariance framework), and test possible differences in the underlying construct. However, illustration of the full potential of factor analytic techniques was outside the scope of the present paper.

There are a number of limitations to this CTT demonstration. First, PROMIS items were developed within a modern test theory framework, i.e. IRT; hence, these items were developed in a way that they are suitable to be administered as part of computerized-adaptive testing (CAT). Thus, item redundancy would not be observed in case of CAT, as only a subset of items would be administered. Also, any floor effects would be mitigated in practice, as the most severe items would be administered only to the most severe subjects. Therefore, the application of CTT methods in general to an instrument that was developed for and intended to be used with modern test theory methods is somewhat limited but applied here for illustrative purposes to compare three different test theory methods (CTT, IRT, RMT). Moreover, the illustration of CTT methods was limited in the sense that many more types of reliability and validity could have been considered. For example, alternative reliability tests include parallel-tests, split-half reliability, test-retest reliability, inter-rater reliability, etc. [17]. Internal consistency may also be investigated by inspecting inter-item correlational patterns or by using Structural Equation Modeling [44]. Additionally, construct validity may subsume convergent, discriminative, and predictive validity. Convergent and predictive validity are usually explored by investigating the associations between instrument scores and some gold standard. However, these types of gold standards are generally not available for PRO measures [45]. In the present case, the dataset that was distributed to the research teams lacked concurrent measures that could be used for assessing this type of validity and we also lacked a variable to identify subjects in the sample, for example, those with a clinical depression diagnosis; hence, these types of analyses were not possible and also beyond the scope of such paper. Finally, because this demonstration was conducted on an item bank rather than a static instrument, measurement properties were only explored at the item-level because the items are not intended to all be used to produce a score. When items can be combined to produce a domain score, CTT can be used to evaluate if the resulting scores are reliable, valid. Nevertheless, within the limits of a scientific paper aimed at giving an introduction to and illustration of a practical application of a test theory method such as CTT, it is hoped that this paper still succeeded in giving a sufficiently comprehensive overview of classical test theory methods.

## Conclusions

The results of the psychometric analyses were considered against the conceptual framework as identified by the developers of the item bank. Overall, the items of the PROMIS Depression Item Bank performed well and seemed appropriate for measuring the unidimensional construct of depression. It is our hope that the illustration of CTT methods in this paper will help researchers to evaluate item- and scale-performance of PRO instruments. Among the advantages of CTT methods are that they are relatively easy to understand and apply, and that statistical software to perform the analyses is widely available. In conclusion, a combination of both classical and modern test theory methods will not only help evaluate the quality of a PRO instrument but may eventually also help advance the assessment and interpretation of psychological constructs that these instruments intend to measure.

## Data Availability

The datasets supporting the conclusions of this article are available from the PROMIS Health Organization, http://www.healthmeasures.net/explore-measurement-systems/promis

## Abbreviations

CAT: Computerized adaptive testing
CFA: Confirmatory factor analysis
CFI: Comparative fit index
CTT: Classical test theory
IRT: Item response theory
ISOQOL: International Society for Quality of Life Research
PRO: Patient-reported outcome
PROMIS: Patient-reported Outcomes Measurement Information System
RMSEA: Root mean square error of approximation
RMT: Rasch measurement theory
SIG: Special Interest Group
TLI: Tucker Lewis index
WLSMV: Weighted least squares means and variance adjusted
